# Preparedness and response to the international poliovirus and diphtheria reintroduction alert: public health interventions and strategy review in the Veneto Region, Italy

**DOI:** 10.3389/fpubh.2025.1510785

**Published:** 2025-06-05

**Authors:** Marco Milani, Michele Nicoletti, Michele Tonon, Davide Gentili, Stefan-Alexandru Panaite, Filippo Da Re, Andrea Basso, Gloria Pagin, Marco Zampini, Debora Ballarin, Francesca Zanella, Vanessa Groppi, Silvia Cocchio, Francesca Russo, Vincenzo Baldo

**Affiliations:** ^1^Department of Cardiac, Thoracic and Vascular Sciences, School of Medicine and Surgery, University of Padua, Padua, Italy; ^2^Regional Directorate of Prevention, Food Safety, Veterinary Public Health- Regione del Veneto, Venice, Italy; ^3^Environmental Prevention and Protection Agency of Veneto Region, Padova, Italy

**Keywords:** poliomyelitis, diphtheria, surveillance, public health, vaccine preventable disease, wastewater surveillance, vaccination strategies

## Abstract

**Background:**

Diphtheria and polio continue to pose significant public health challenges globally, making sustained high vaccination coverage crucial. This study examines the strategies adopted in the Veneto Region to enhance vaccination rates for diphtheria and polio among priority target groups and improve poliovirus surveillance, following the WHO alert about the potential reintroduction and circulation of the two pathogens.

**Aim:**

The main objective of this study is to assess the effectiveness of supplementary vaccination strategies implemented in the Veneto Region in response to international poliomyelitis and diphtheria alerts. Additionally, it aims to identify municipalities at higher risk of poliovirus AFP based on georeferenced vaccination coverage, enhancing environmental surveillance strategies. Ultimately, the study evaluates regional preparedness and response efforts, offering insights to mitigate the risk of reintroducing these diseases and providing a framework adaptable to similar contexts.

**Methods:**

The Regional Directorate of Prevention, Food Safety, Veterinary Public Health analysed regional vaccination coverage and provided Local Health Authorities (LHAs) with a georeferenced representation of vaccination coverage across municipalities. Directives on targeted vaccination strategies were issued to address identified gaps and improve readiness. Ten months later, the Regional Directorate assessed the approaches implemented by LHAs to improve vaccine uptake and evaluated the resulting vaccination coverage. Based on this georeferenced analysis, the effectiveness of current environmental poliovirus surveillance was reviewed, and recommendations for optimising surveillance efforts were proposed.

**Results:**

Following the implementation of the vaccination strategies recommended by the Regional Directorate, vaccination rates improved across all LHAs, especially among children aged 0–2 years who had not received any vaccine doses. The georeferenced analysis of vaccination coverage revealed critical gaps in environmental poliovirus surveillance and underscored the need for targeted interventions to reach unvaccinated populations.

**Conclusion:**

This study demonstrates that centralised governance, combined with georeferenced vaccination data, environmental poliovirus surveillance, and clinical AFP surveillance, enhances the ability to identify coverage gaps and respond to infectious disease threats. While improvements in vaccination rates were achieved, the findings underscore the need for targeted, community-specific interventions and continuous monitoring to address disparities. Strengthening data integration and adopting innovative surveillance methods will be crucial to sustaining high vaccination coverage and ensuring public health security.

## Introduction

During 2022, the World Health Organization (WHO) issued alerts regarding the potential reintroduction of poliovirus in European countries that had previously achieved polio-free status (1). This concern stemmed from the detection of vaccine derived poliovirus (cVDPV) in wastewater in regions such as the United Kingdom, the United States, and Israel, highlighting the risk of international spread to EU/EEA countries (2). Notably, wild poliovirus type 1 (WPV1) is still endemic in Afghanistan and Pakistan, where ongoing transmission challenges eradication efforts. Additionally, outbreaks of cVDPV have been reported in over 30 countries, including regions in Africa, the Middle East, and Southeast Asia, reflecting gaps in immunization coverage and surveillance systems (3). Concurrently, the European Centre for Disease Prevention and Control (ECDC) reported a fivefold increase in diphtheria cases from January 2022 to August 2023 compared to the 2017–2021 average, emphasizing the renewed threat posed by this disease (4). Remarkably, 391 cases were reported across nine countries (Austria, Czechia, France, Germany, Italy, Netherlands, Norway, Switzerland, and the United Kingdom), with the majority occurring during the second half of 2022 (5). Amid these international developments, several social and epidemiological factors have increased the risk of epidemics, including intensified migratory flows from regions where these diseases remain endemic, disruptions in vaccination programs due to the COVID-19 pandemic, and rising vaccine hesitancy (6–9).

In Italy, despite mandatory vaccination introduced by National Law n.119/2017, coverage remains suboptimal in certain regions and among specific population groups, leaving gaps in herd immunity (10–12). Moreover, while inactivated poliovirus vaccine (IPV) effectively prevents disease, it does not confer mucosal immunity in the gastrointestinal tract. Consequently, vaccinated individuals can still become infected and shed the virus through faeces, facilitating its circulation and transmission within the community (13). To address these risks, the Veneto Region implemented over the years a series of public health interventions. The Veneto Region expanded the national acute flaccid paralysis (AFP) surveillance system, originally implemented in 1995, by including individuals over the age of 15 in addition to the national recommendation of monitoring those under 15 years of age (14). Furthermore, since 2008, the Regional Directorate of Prevention, Food Safety, Veterinary Public Health has actively monitored regional vaccine coverage to identify gaps and ensure timely interventions, in alignment with national provisions.

In light of the recent international development, the Italian Ministry of Health (MoH) called upon Regional Authorities to strengthen preparedness and response. Indeed, in Italy, the national health service (SSN) is coordinated centrally, while operational capacity is entrusted to regional authorities. The Veneto Region, with a population of 4,905,854 inhabitants and located in the northeast of Italy, maintains robust central governance over public health matters through the Regional Directorate of Prevention, Food Safety, and Veterinary Services, which oversees nine Local Health Authorities (LHAs). LHAs are decentralized public health entities responsible for delivering healthcare services and implementing public health measures within their designated geographic areas.

Given these challenges, the main objective of this study is to assess the effectiveness of supplementary vaccination measures implemented in the Veneto Region in response to international poliomyelitis and diphtheria alerts, through a georeferenced analysis of vaccination coverage across the region’s municipalities.

A secondary aim is to identify municipalities at higher risk of poliovirus AFP, based on georeferenced vaccination coverage data, with the goal of optimising environmental surveillance strategies.

The ultimate aim of this analysis is to provide a comprehensive evaluation of regional preparedness and response efforts, offering critical insights into strategies to effectively mitigate the risk of reintroducing these infectious diseases. The approaches and findings presented in this study offer a framework that can be adapted and implemented in other regions facing similar challenges.

## Materials and methods

### Study design

In response to the international poliovirus and diphtheria alerts, the Regional Directorate of Prevention, Food Safety, and Veterinary Public Health implemented a mixed-methods strategy. This strategy combined monitoring and enhancing routine immunization campaigns, strengthening environmental surveillance to detect poliovirus in wastewater, and improving clinical networks for the early detection of acute flaccid paralysis (AFP) cases. The Regional Directorate, which oversees and coordinates public health policies at the regional level, provided guidance and support to Local Health Authorities (LHAs). Additionally, the Regional Directorate evaluated the implementation and effectiveness of specific interventions adopted by the LHAs to improve vaccination coverage and mitigate disease risks. This comprehensive approach enabled an integrated assessment of clinical, environmental, and strategic measures to maintain polio-free status and prevent diphtheria resurgence in the region.

### Monitoring and enhancing immunization

On September 21, 2022, in response to national guidelines, the Regional Directorate issued a directive to all Local Health Authorities (LHAs) aimed at reducing the risk of poliovirus and diphtheria reintroduction (14). This directive, aligned with the strategic objectives set by the World Health Organization (WHO) and the Italian Ministry of Health, recommended a series of targeted measures, including achieving high vaccination coverage alongside other public health interventions, as outlined in Table 1. The directive further informed LHAs that a regional analysis would be conducted and shared with them to support the effective implementation of these interventions.

**Table 1:** Strategic objectives and priority interventions stated by the Regional Directorate of Public Health.

Strategic objectives	Actions	Expected results
Maintain vaccination coverage of newborns, infants, and adolescents above 95% in each district, municipality, and community, to minimise the risk and consequences of poliovirus and diphtheria circulation	Analysis of detailed georeferenced data on vaccination coverage for polio and diphtheria among individuals under 18 years (cohort 2004, performed by the Regional Directorate and disseminated to LHAs). Identification of priority interventions in municipalities/areas with higher vaccinal gaps. Active solicitation of all children under 7 years of age without a complete vaccine cycle in municipalities with coverage below 95%. Reinforce recommendations and counselling for polio and diphtheria vaccinations by Primary Care Paediatricians/General Practitioners (GPs) to unvaccinated individuals under 18 years old. Ensure vaccination accessibility for polio and diphtheria also at the COVID-19 vaccination clinics.	Enhancement of specific vaccination coverage
Enhance surveillance of Acute Flaccid Paralysis	Advise the clinical network of Acute Flaccid Paralysis surveillance to promptly respond to all activities.	Prompt detection of AFP that could be related to poliovirus infection
Strengthen environmental surveillance of poliovirus	Assessment of the regional environmental surveillance of poliovirus in wastewater.	Improve early warning
Enhance dTap vaccination among pregnant women	A specific recommendation was issued to all healthcare personnel involved in the maternity care pathway to actively promote the recommended vaccinations for pregnant women, including those for diphtheria, tetanus, pertussis, and influenza. It is specifically advised that this recommendation be documented in the reports of check-up visits conducted by the gynaecologist. This action aims to address potential risks that may be unclear to mothers and, importantly, to all healthcare personnel.	Reduce diphtheria risk for newborn
Enhance vaccination of travellers visiting areas with poliovirus circulation	Recommend travel Medicine clinic operators, General Practitioners, and Primary Care Paediatricians to check the vaccination status for polio and diphtheria in case of travel to high-risk countries, considering CDC guidelines: “*For adults with a primary series and a booster dose with IPV vaccine travelling to a high-risk country for more than 4 weeks, for whom the last vaccine dose was administered more than 12 months before the departure date, consider administering an additional IPV booster dose.*”	Reduce risk among international travellers and avoid imported cases
Enhance vaccination of migrants, refugees, and asylum seekers	Verify vaccination intakes and related coverage, activating *ad hoc* interventions where appropriate, involving cultural mediators (also with on-site vaccination initiatives), with priority in more critical contexts.	Reduce risk among hard to reach population
Strengthen the response capacity of healthcare workers	To ensure correct isolation procedures, verify and update all LHAs and Hospital protocols for managing suspected, probable, or confirmed polio and diphtheria cases.	Increase awareness in healthcare workers

In order to obtain a comprehensive and geographically detailed assessment of poliovirus and diphtheria vaccination coverage, the Regional Directorate extracted data from the regional digital vaccination registry (SIAVr) on October 1, 2022. SIAVr is a centralized immunization database, which allows for precise and timely monitoring of vaccination activities and outcomes. Using Microsoft Excel 2013 and R 4.3.0, the data were aggregated at the municipal level and analysed by year of birth. The analysis focused on three groups: children aged 1 to 2 years (born 2021–2020) who had received at least the first dose of anti-polio and anti-diphtheria vaccine; children aged 3 to 7 years (born 2019–2015) who had completed the three-dose primary vaccination cycle; and individuals aged 8 to 18 years (born 2014–2004) who had received at least four doses, including the primary cycle and the first booster. On October 11, 2022, the Regional Directorate disseminated the results of the vaccination coverage analysis to the Local Health Authorities (LHAs), providing detailed breakdowns by geographic area and age group. The Directorate reiterated the strategies outlined in the earlier regional directive and introduced additional measures to address specific gaps. These new indications included verifying the adequacy of fourth-dose coverage among individuals under the age of 18 in municipalities where coverage was below 90%, providing general practitioners and paediatricians with detailed information on local vaccination coverage and epidemiological risks, and assessing vaccination status during other scheduled immunizations, such as those against COVID-19 or influenza. Additional measures also emphasized ensuring that operators in migrant reception centres were adequately immunized.

LHAs were instructed to use the provided data to identify local areas and populations with low vaccination coverage. They were encouraged to adopt tailored, context-specific approaches based on, but not limited to, the recommended strategies outlined by the Region.

### Immunization strategies evaluation

To assess the effectiveness of the immunization enhancement strategies, a follow-up evaluation was conducted on July 24, 2023. The Regional Directorate distributed a structured survey (Supplementary 1) to the LHAs via Google Forms, collecting detailed information on the regionally recommended measures they had implemented.

The survey first inquired whether the LHAs had initiated specific vaccine recovery strategies targeting diphtheria and poliomyelitis, requesting details on when such measures began, which age groups were prioritized, and what tactics were used to increase vaccination uptake. The survey included questions on data cleansing, quality assessment, and optimization procedures, such as recovering missing or incomplete vaccination records, verifying that individuals listed in the registries are still residing in the region, and updating digital vaccination registries to achieve a more accurate representation of coverage. It asked whether LHAs employed active solicitation efforts, including direct invitations, scheduled appointments, and the option to reschedule for those not fully immunized. The survey also explored whether LHAs offered walk-in sessions, allowing citizens to receive vaccinations without an appointment during predefined time slots at dedicated healthcare facilities. Finally, it examined the involvement of general practitioners and primary care clinics, considering their role in informing their served populations, issuing active invitations through phone calls or SMS, and, where applicable, directly administering vaccinations. It also asked if and how LHAs monitored their recovery campaigns and what challenges they encountered during implementation.

Subsequently, on September 1, 2023, vaccination coverage data were re-extracted and analysed using the same criteria as the initial assessment. This evaluation allowed for a direct comparison of vaccination rates before and after the implementation of the strategies, thereby highlighting any improvements achieved, measuring the overall effectiveness of the implemented strategies, and identifying persistent gaps requiring further attention. Additionally, data was analysed to discern whether newly vaccinated individuals were originally non-immunised or only partially immunised.

### Environmental surveillance

In response to the ECDC alert highlighting the circulation of vaccine-derived poliovirus in the European Region in 2022, the Regional Directorate introduced a poliovirus environmental surveillance in collaboration with the Regional Agency for the Environment (ARPAV), the official Veneto Regional Agency responsible for monitoring and safeguarding environmental quality. The program involved regular sampling and testing of wastewater at multiple sites across the region. The specific locations where surveillance was activated are shown in Supplementary 2. Each selected wastewater treatment plant represented a basin serving between 50,000 and 150,000 citizens. Samples consisted of sewage effluent collected at the entrance of the treatment plants. Details on the processing and analysis procedures are provided in Supplementary 3. The geospatial presentation of data was developed using the Free and Open Source QGIS, version 3.30.1, using the ISTAT (the Italian National Institute of Statistics) layer for municipal boundary delineation and the Veneto Region layer for delineating the territory of each LHA.

### Acute flaccid paralysis surveillance

The national AFP surveillance system entails the reporting, sample collection, and follow-up of all AFP cases under 15 years of age (13). Reporting is facilitated through a network of 130 physicians spanning Paediatric, Neurology, Infectious Diseases, and Intensive Care units across every hospital and academic centre in the region. The network acts as a sentinel system, ensuring prompt reporting of AFP to the Regional Directorate, which forwards the data to the Italian Ministry of Health (MoH) and the National Institute of Health (ISS). Notifications are submitted within 48 h to 7 days of symptom onset. For AFP cases in individuals over 15 years of age, the same reporting network is used; however, data are not forwarded to the MoH and ISS, as this surveillance is conducted at the regional level. For all AFP cases, two stool samples are collected within 14 days of symptoms onset and sent to accredited laboratories for virological investigations and diagnosis confirmation. Additionally, clinical evaluations are conducted 60 days post-onset to document any residual paralysis. In addition to routine reporting, the Regional Directorate engages with designated LHA contacts every 15 days to closely monitor AFP case numbers and reinforce follow-up evaluations for previously reported cases.

### Clinical diphtheria surveillance

As part of the regional directive, physicians and healthcare facilities were required to strengthen clinical surveillance for diphtheria, with instructions to monitor for both respiratory and cutaneous forms of the disease. The directive mandated heightened vigilance in identifying clinical presentations indicative of diphtheria and ensured the prompt reporting of any suspected cases to public health authorities. These measures were designed to enable timely investigation, accurate diagnosis, and immediate implementation of appropriate public health interventions.

## Results

On September 1, 2023, the survey on the strategies adopted by LHAs to increase vaccination coverage for diphtheria and polio among priority target groups was completed. Strategies adopted by each LHA, stratified by type of intervention and targeted age group (I: 1–2 years; II: 3–7 years; III: 8–18 years) are reported in Supplementary 4. According to the survey results, 8 out of 9 LHAs verified their territorial coverage and implemented data cleansing, quality assessment, and optimization for all age groups. An active solicitation strategy was adopted by all LHAs for at least 1 of the 3 age groups considered: 8 out of 9 for group I, 7 out of 9 for group II, and 7 out of 9 for group III. Walk-in vaccination without an appointment was offered by 4 LHAs for groups I and II, and by 3 LHAs for group III. Finally, the involvement of General Practitioners (GPs) was undertaken by 6 LHAs for group I, and by 5 LHAs for groups II and III.

**Figure 1:**
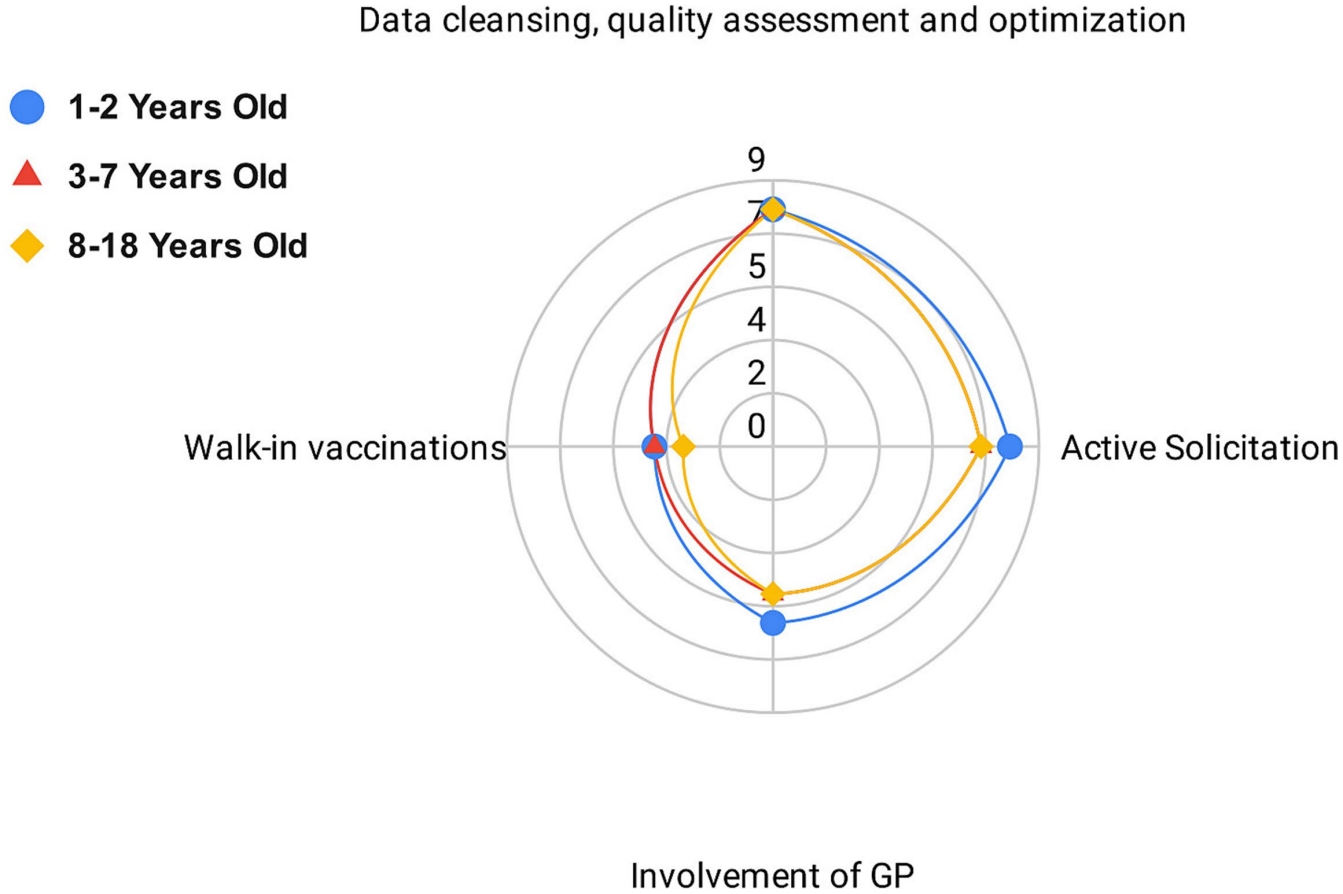
Number of local health authorities adopting each vaccine retrieval strategy grouped by identified criteria.

The strategies adopted by all LHAs within the three age groups are shown in Figure 1.

Data cleansing, quality assessment and optimisation, along with active solicitation, were the most commonly implemented strategies across all age groups. These were followed by the involvement of general practitioners (GPs) and the offering of walk-in vaccination services.

When examining active solicitation strategies, Supplementary 5 (S5) summarizes the specific methods used by each LHA to contact the families of unvaccinated or partially vaccinated children, specifying whether outreach was conducted via postal letter, phone call, or text message (SMS). The Supplementary Table S5 also indicates whether vaccinations offered through this outreach were scheduled as fixed appointments or provided as walk-in services without an appointment. The primary active solicitation strategy, adopted by 8 out of 9 LHAs, was postal letters. Additionally, 3 LHAs used text messages (SMS) and 4 LHAs made telephone calls. In 7 out of 9 LHAs, appointments were scheduled through telephone and/or postal contact, while in 2 LHAs, active solicitation informed citizens of the possibility of walk-in vaccination without an appointment.

Table 2 reports the variation in polio and diphtheria vaccination coverage pre- and post-intervention for Polio and Diphtheria across different LHAs.

**Table 2:** Polio and diphtheria vaccination coverage pre- and post-vaccine retrieval intervention, divided by local health authorities (LHAs), and grouped as per criteria.

LHAs	Group	Vaccination coverage		
		Pre	Post	Delta
A	I^*^	95.7%	96.4%	0.7%
	II^**^	94.6%	95.1%	0.6%
	III^***^	92.9%	93.1%	0.2%
B	I	95.5%	96.6%	1.2%
	II	94.9%	95.5%	0.7%
	III	91.8%	92.0%	0.2%
C	I	96.3%	96.7%	0.3%
	II	94.6%	95.4%	0.8%
	III	90.6%	91.0%	0.4%
D	I	95.6%	96.7%	1.1%
	II	94.5%	96.1%	1.5%
	III	93.1%	94.0%	0.9%
E	I	95.6%	96.5%	0.9%
	II	93.9%	95.5%	1.6%
	III	90.4%	92.0%	1.6%
F	I	95.5%	96.0%	0.5%
	II	94.3%	94.8%	0.5%
	III	90.8%	91.1%	0.3%
G	I	94.0%	94.9%	0.9%
	II	93.0%	93.5%	0.5%
	III	88.6%	88.8%	0.2%
H	I	95.7%	96.3%	0.6%
	II	94.5%	94.8%	0.3%
	III	91.2%	91.2%	0.0%
I	I	97.0%	97.3%	0.3%
	II	95.7%	96.3%	0.6%
	III	92.7%	93.3%	0.6%
Veneto Region	I	95.8%	96.5%	0.7%
	II	94.7%	95.3%	0.6%
	III	91.3%	91.7%	0.4%

* Born in 2021–2020 (1–2 years old): vaccinated with at least the 1st dose.

** Born in 2019–2015 (3–7 years): vaccinated with at least the 3rd dose (completion of the primary cycle).

*** Born in 2014–2004 (8–18 years): vaccinated with at least the 4th dose (primary cycle and 1st booster dose).

**Figure 2:**
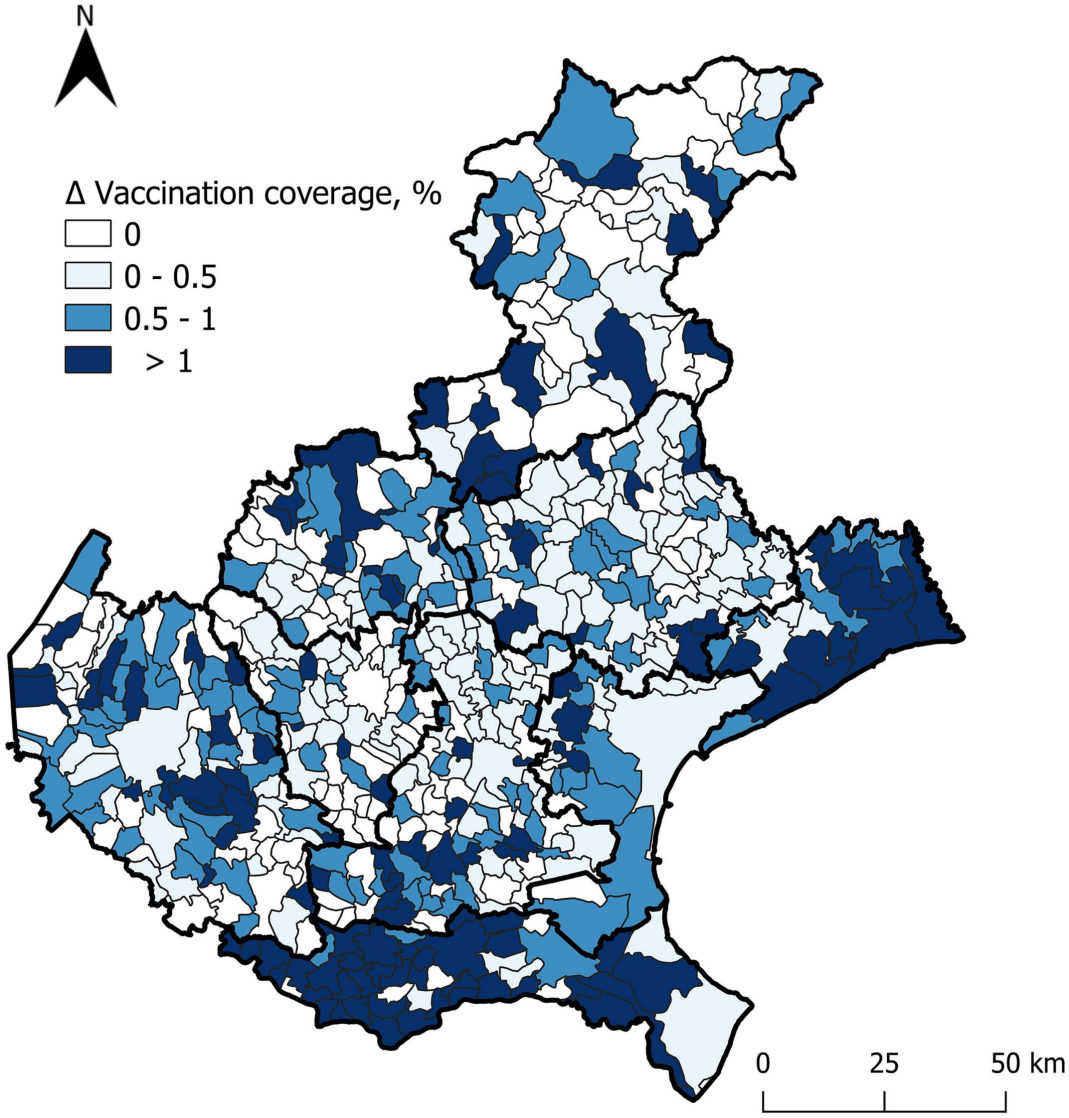
Positive percentage differences in poliovirus and diphtheria vaccination coverage before and after the intervention for individuals aged 1 to 18 years (cohorts 2021–2004), across municipalities.

Since polio and diphtheria vaccines are usually combined or co-administered, data regarding vaccination coverage for these two pathogens are overlapping. Similarly, the changes in vaccination coverage across the three age groups showed identical patterns for both vaccines. In group I, coverage increased by an average of 0.7% (range 0.3–1.2%). For group II, the average increase was 0.7% (range 0.3–1.6%). Lastly, in group III, there was an average coverage increase of 0.4% (range 0–1.6%). Georeferenced data on diphtheria and polio vaccination coverage among birth cohorts from 2004 to 2021, which were included in the catch-up vaccination regional recommendation, are presented in Supplementary 6 for both pre- and post-intervention. Figure 2 reports the percentage variation in poliovirus and diphtheria vaccination coverage for each municipality, serving as the sole proxy.

The greatest percentage increases occurred in areas where initial coverage was below 95%. Particularly noteworthy is the increase in vaccination coverage observed among individuals who had not yet initiated the national vaccination schedule, especially in the southeastern regions. However, suboptimal coverage persists in isolated areas in the central region.

**Figure 3:**
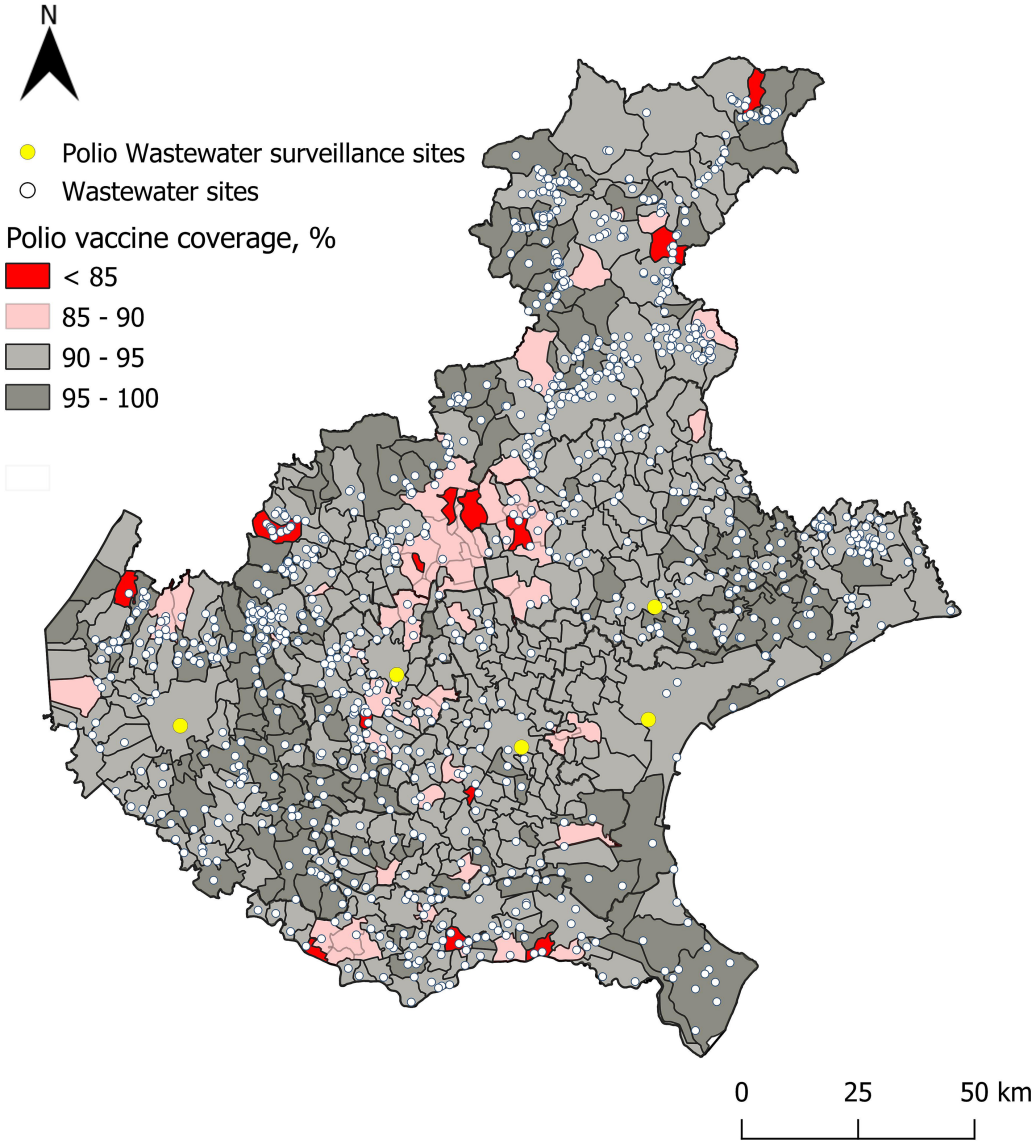
Wastewaters sampling sites and active surveillance sites, based on actual polio vaccination coverage values in the Veneto Region.

With the updated data post-catch-up action, Figure 3 displays currently active wastewater sampling sites and updated polio vaccination coverage values across municipalities.

During 2022, following the strategic objectives provided by the Regional Directorate, an increased number of flaccid paralyses was recorded, as shown in Supplementary 7. The leading clinical diagnosis of acute flaccid paralysis were Guillain-Barré Syndrome (GBS) and its Miller Fisher variant, accounting for 37% of cases. Other diagnoses included neuropathy (17%), viral meningoencephalitis (15%), acute transverse myelitis (4%), and spinal compression (3%). The aetiology was not provided in 24% of cases involving adults, as follow-up for adults is not mandatory. Among these cases, none were associated with poliovirus, consistent with the environmental wastewater surveillance.

## Discussion

This study represents a rapid and coordinated response to international alerts issued by the WHO and ECDC, which identified the circulation of vaccine-derived poliovirus in the European region in 2022 and highlighted the potential risk of diphtheria dissemination. The findings underscore the advantages of integrating multiple surveillance systems, including vaccination coverage monitoring and clinical surveillance for both poliovirus and diphtheria cases. Additionally, environmental surveillance, as conducted in the specific case of poliovirus, serves as a critical tool for early detection of potential viral circulation and complements the broader public health strategy. The synergistic use of these data sources not only strengthens the effectiveness of each surveillance component but also equips the Regional Directorate with critical insights, enabling evidence-based governance and timely, targeted interventions to mitigate risks associated with these vaccine-preventable diseases.

When compared with other regions in Italy, Veneto consistently achieves vaccination coverage rates above the national average across all age groups. For instance, in 2022, the coverage rate for polio (95.45%) and diphtheria (95.46%) among 2-year-old children was higher than the national average of 95.15% for polio and 95.14% for diphtheria, respectively. Similarly, at ages 8 and 18, the region maintains superior coverage rates for these antigens compared to national figures, further demonstrating the robustness of its vaccination programs (15). Nevertheless, the study reveals significant disparities across the municipalities of the region. These disparities can be partially attributed to the presence of active anti-vaccination movements, reflecting the historical presence of vaccine resistance rooted in specific regional territories, which foster localised resistance and reduce vaccination uptake (16). These gaps create localized vulnerabilities, particularly concerning the potential reintroduction of poliovirus and diphtheria (17–20). Such variability underscores the necessity of adopting a granular, community-specific approach to vaccination strategies. Localized analyses, conducted by each LHA, can further identify areas and population groups where vaccination efforts need strengthening, facilitating the design and implementation of targeted intervention plans to address these gaps effectively (21–23).

Moreover, geospatial analyses of vaccination data can guide efforts to optimize the selection of environmental sampling points and strengthen AFP surveillance networks. Our findings suggest that integrating vaccination coverage data into surveillance planning can enhance both the early detection of poliovirus and preparedness for potential AFP cases. While high IPV vaccine coverage does not necessarily prevent virus circulation due to the lack of mucosal immunity, directing surveillance efforts toward areas with low IPV vaccine coverage can enhance the early detection of poliovirus circulation. This, in turn, allows for timely interventions to mitigate potential AFP outbreaks, particularly among unvaccinated individuals. This targeted approach ensures that resources are allocated efficiently to regions most vulnerable to developing clinical manifestations, thereby maximizing the impact of public health interventions and strengthening early warning systems.

This study supports that a vaccination mandate alone is insufficient to ensure consistent high coverage across all communities. To address this, structured interventions are essential. Our findings indicate that when Local Health Authorities (LHAs) employed strategies such as territorial and community assessments, coverage audits, and recovery initiatives, increases in vaccination coverage were observed. This aligns with existing evidence that vaccination strategies, particularly those involving active outreach and personalized communication, are effective in improving vaccine uptake (24). Structured interventions, such as the ones mentioned, are designed to identify and reach unvaccinated or partially vaccinated populations. Active solicitation through personalized communication, such as letters, phone calls, and targeted outreach by general practitioners and paediatricians, plays a crucial role in increasing vaccine uptake (25–27). These efforts leverage the trusted relationships between healthcare providers and patients to improve compliance and enhance the effectiveness of vaccination campaigns (28, 29). Moreover, as robustly evidenced in the literature, effective communication strategies are critical for addressing the dynamics of emotional epidemiology, where public risk perception plays a decisive role in vaccine uptake (30–34). Paradoxically, the success of vaccination programs in eradicating diseases such as polio has inadvertently diminished perceived risk among the population (35). This complacency underscores the need for strategic advocacy campaigns to remind individuals of the persistent threat posed by seemingly dormant pathogens. These efforts are particularly vital in an era characterized by increased migration and international travel, which amplify the risk of disease reintroduction and potential outbreaks.

The ECDC’s identification of diphtheria clusters within migrant populations in Europe further highlights the necessity of a multifaceted approach to public health. Targeted vaccination campaigns are essential to reach at-risk and hard-to-reach populations, such as migrants, refugees, and underserved communities. Proactive strategies—including collaboration with cultural mediators, on-site vaccination initiatives, and personalized outreach—can effectively engage these groups and reduce barriers to vaccine access (36–38). The effectiveness of these approaches is supported by evidence highlighting the importance of community engagement, trust-building, and culturally appropriate interventions to improve participation among socially disadvantaged groups. Additionally, flexible delivery models, such as offering vaccinations at community centres or during home visits, can help overcome logistical challenges and improve access for populations who may face difficulties attending traditional healthcare settings (39, 40).

Integrating these targeted vaccination efforts with enhanced clinical surveillance can prove to be successful for early detection and timely intervention. A robust surveillance system can identify diseases such as acute flaccid paralysis (AFP) for poliovirus or respiratory symptoms for diphtheria, particularly in regions with identified coverage gaps. This strengthens the capacity to detect and respond to potential outbreaks. The extension of clinical AFP surveillance in the Veneto Region to include individuals over 15 years old could further improve the detection of virus circulation. This is especially relevant in Italy, where the Sabin vaccine (oral poliovirus vaccine), which provides mucosal immunity, has not been used for over 30 years. In such contexts, adults may have low mucosal immunity, allowing virus transmission even among vaccinated individuals (41). Although no cases of poliovirus-related AFP were detected in this study, the extended surveillance system for adults remains a valuable tool for early detection and timely intervention. This approach is supported by real-world evidence, such as the 2022 case in Rockland County, New York, where an unvaccinated adult developed paralytic polio in a polio-free country (42).

At a national level, centralized governance of vaccination recovery efforts plays a pivotal role in achieving uniform outcomes by ensuring that all regions align their activities with clearly defined common objectives. In the Veneto Region, regional initiatives have significantly improved vaccination coverage across all LHAs. This demonstrates not only the effectiveness of targeted vaccination strategies but also the importance of strong governance and coordination at the regional level. A structured vaccination framework enables regions to implement consistent strategies across diverse healthcare contexts, share best practices, and address specific local challenges effectively. In the Veneto Region, the successful implementation of national directives illustrates how dialogue and coordination among stakeholders can lead to measurable improvements in vaccine coverage. By fostering this collaboration, centralized governance enhances the efficiency and equity of vaccination efforts. Moreover, it strengthens the resilience of public health systems, enabling them to effectively tackle both current and future immunization challenges.

This study demonstrates that integrating geospatial vaccination coverage, environmental poliovirus monitoring, and clinical AFP surveillance enhances public health preparedness, and provides a highly replicable framework for other regions and countries. While vaccination mandates are important, structured interventions like targeted outreach and community-specific assessments are essential for achieving consistent coverage. Extending AFP surveillance to adults supports early detection of poliovirus circulation. These integrated efforts provide policymakers with critical insights to guide evidence-based decision-making, strengthen public health resilience, and mitigate the risk of vaccine-preventable disease reintroduction.

Despite its strengths, this study has certain limitations. The lack of historical data and record linkage for specific groups, such as pregnant women, refugees, and asylum seekers, hindered a comprehensive assessment of vaccination coverage changes. Additionally, small population sizes in certain municipalities affected the granularity of the graphical representation of vaccination coverage changes. Moreover, the absence of precise mapping data for wastewater catchment basins limited the ability to correlate environmental surveillance data with vaccination coverage fully. Future efforts should focus on addressing these limitations by improving data collection and record linkage mechanisms for vulnerable populations. Expanding the use of geospatial analysis and refining surveillance protocols will further enhance the region’s preparedness for emerging public health threats.

## Conclusion

This study provides evidence that centralised governance interventions play a strategic and effective role in directing preventive activities and responding to infectious disease threats. Furthermore, the integrated surveillance system (georeferenced vaccination coverage, environmental poliovirus monitoring and clinical surveillance) proves to be a valuable tool for enhancing preparedness and readiness to infectious disease alerts.

While achieving high overall vaccination coverage is critical, it must be complemented by detailed local-level analyses and targeted interventions to address disparities within specific communities. Georeferenced vaccination and clinical surveillance data offer valuable tools to guide targeted environmental monitoring and refine public health strategies. However, limitations such as gaps in data for high-risk populations, including pregnant women, refugees, and asylum seekers, and small sample sizes in some areas, highlight the need for improved data integration and expanded surveillance efforts. Moving forward, embracing innovative approaches, such as molecular diagnostic techniques for environmental monitoring, and fostering stronger collaborations with healthcare providers and community leaders will be essential. These integrated efforts will not only help close vaccination gaps but also strengthen health systems, enhance outbreak detection, and ensure equitable access to vaccines for all.

## Data Availability

The raw data supporting the conclusions of this article will be made available by the authors, without undue reservation.
