# Supplementary material for: Preparedness and response to the international poliovirus and diphtheria reintroduction alert: public health interventions and strategy review in the Veneto Region, Italy
Source: Front Public Health. 2025 Jun 5;13:1510785. doi: 10.3389/fpubh.2025.1510785 (PMC12176779; doi:10.3389/fpubh.2025.1510785)
Supplement: Supplementary file 1 [file Data_Sheet_1.docx]

Supplementary Material

| Question | Answer |
| --- | --- |
| Indicate LHA | (indicate LHA) |
| Has your LHA implemented a recovery vaccination coverage plan for Diphtheria and Poliomyelitis? | ◻Yes  ◻ No |
| If so, when has the recovery vaccination coverage plan started? | (indicate month and year) |
| If so, what was the target of the recovery vaccination coverage plan?* | ◻ Newborns (0-24 months)  ◻ Preschool age (2-7 years)  ◻ Children and adolescents (8-18 years) |
| Which strategies were adopted for the Newborns (0-24 months)?* | ◻ Data cleansing, quality assessment and optimization  ◻ Active solicitation  ◻ PLS involvement  ◻ GP involvement  ◻ Walk-in vaccination without appointment  ◻ No strategy |
| Which strategies were adopted for Preschool age (2-6 years)?* | ◻ Data cleansing, quality assessment and optimization  ◻ Active solicitation  ◻ PLS involvement  ◻ GP involvement  ◻ Walk-in vaccination without appointment  ◻ No strategy |
| Which strategies were adopted for Children and adolescents (7-18 years)?* | ◻ Data cleansing, quality assessment and optimization  ◻ Active solicitation  ◻ PLS involvement  ◻ GP involvement  ◻ Walk-in vaccination without appointment  ◻ No strategy |
| If so, how were PLSs and GPs involved? | (free text) |
| If an active call was made, what mode was used?* | ◻ Phone call  ◻ Postal letter  ◻ Text message (SMS) |
| In the event that the recovery involved the active call, this included: | ◻ Walk-in vaccination without appointment  ◻ Appointment |
| Has LHA monitored the recovery campaign? | ◻ Yes  ◻ No |
| If so, how was it structured? | (short free text) |
| What difficulties were encountered? | (free text) |

* It is possible to specify one or more options

**S1. Survey on vaccination coverage recovery plans adopted by each Local Health Authority (LHA)**.


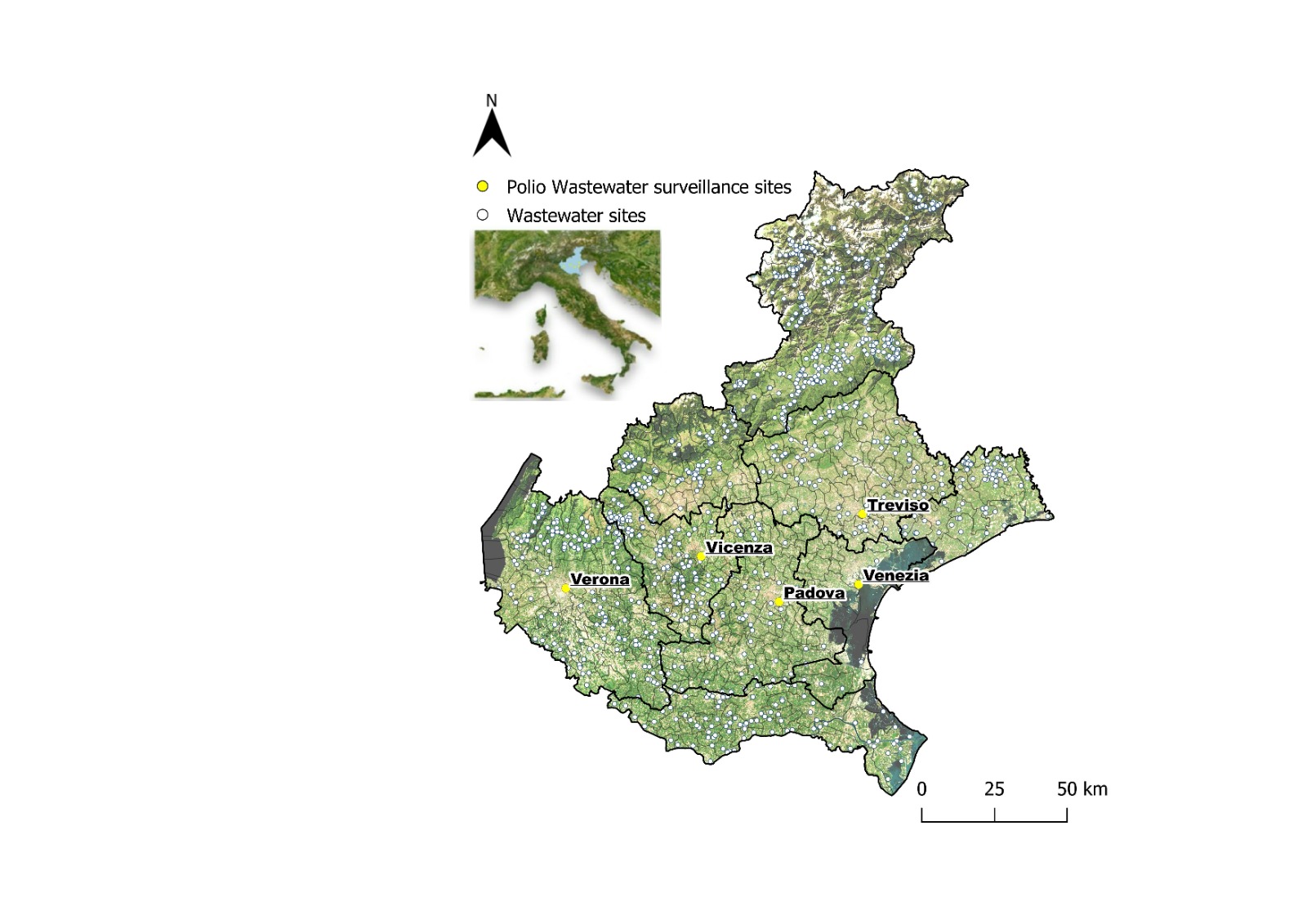


**S2. Wastewater monitoring sites for poliovirus surveillance in the Veneto Region**

This supplementary material provides details on the wastewater monitoring sites across different Local Health Authorities (LHAs) in the Veneto Region and on the active sites selected for poliovirus monitoring. Sampling sites were identified considering various key factors, such as high resident population number and density, significant visitor influx (with tens of millions of tourists every year), and the presence of airports and ports facilitating international travel.

**S3. Poliovirus wastewater sampling and analysis procedures**

Wastewater sampling was conducted using automatic samplers that collected samples proportionally to the water flow variation over a 24-hour period to avoid alterations in the sample. The collected samples were sent to the Regional Agency for the Environment, which was responsible for the purification, concentration, and freezing procedures.

For each sample, 500mL of wastewater was clarified by centrifugation at 4°C. the resulting pellet was stored at 4°C, while the supernatant was concentrated by biphasic precipitation with two polymers, dextran and polyethylene glycol, and a 5N sodium chloride solution. The mixture was subjected to agitation, poured into suitable funnels and allowed to settle overnight, resulting in the formation of three distinct phases. The lower phase and interphase were collected and combined with the pellet from the initial centrifugation. This mixture was treated twice with chloroform to remove bacteria and mold. After shaking and centrifugation, the supernatant was aliquoted and used for inoculation into cell lines.

Isolation of poliovirus and other enteroviruses was performed by the Italian National Institute of Health (ISS) using two cell lines: RD cells, a human rhabdomyosarcoma-derived cell line with abroad spectrum of susceptibility to enteroviruses, and L20B cells, a murine cell line genetically modified by transfection of the receptor for human poliovirus, thus making it susceptible to infection with specific polioviruses and some rare non-polio enteroviruses. The use of both cell lines allows the isolation of polioviruses and other non-polio enteroviruses, from both animals and humans, as well as other enteric viruses, such as Adenovirus or Reovirus.

|  | Data cleansing, quality assessment and optimization | | | Active  solicitation | | | Walk-in vaccination without an appointment | | | Involvement  of GPs | | |
| --- | --- | --- | --- | --- | --- | --- | --- | --- | --- | --- | --- | --- |
| LHA | Age Group | | | Age Group | | | Age Group | | | Age Group | | |
|  | I* | II** | III*** | I | II | III | I | II | III | I | II | III |
| A | x | x | x | x | x | x |  |  |  | x |  |  |
| B | x | x | x | x | x |  | x | x | x | x | x | x |
| C | x | x | x | x | x | x | x | x |  |  |  |  |
| D | x | x | x | x | x | x |  |  |  | x | x | x |
| E | x | x | x | x | x | x | x | x | x | x | x | x |
| F | x | x | x | x | x | x |  |  |  |  |  |  |
| G |  |  |  |  |  | x |  |  |  |  |  |  |
| H | x | x | x | x |  |  | x | x | x | x | x | x |
| I | x | x | x | x | x | x |  |  |  | x | x | x |

* Born in 2021-2020 (1-2 years old): vaccinated with at least the 1st dose

** Born in 2019-2015 (3-7 years): vaccinated with at least the 3rd dose (completion of the primary cycle).

*** Born in 2014-2004 (8-18 years): vaccinated with at least the 4th dose (primary cycle and 1st booster dose).

**S4. Vaccination strategies adopted by all 9 Local Health Authorities (LHAs) by different age groups**.

| Local Health Authorities | Solicitation strategies | Access Modality for Vaccination after Active Outreach |
| --- | --- | --- |
| A | Phone call  Postal letter | Walk-in vaccination  without appointment |
|  | Postal letter | Walk-in vaccination  without appointment |
| C | Postal letter | Appointment |
| D | Phone call  Postal letter | Appointment |
| E | Phone call  Postal letter  Text message (SMS) | Appointment |
| F | Postal letter  Text message (SMS) | Appointment |
| G | Postal letter  Text message (SMS) | Appointment |
| H | Postal letter | Appointment |
| I | Phone call | Appointment |

**S5. Solicitation and access strategies for vaccination among Local Health Authorities.**


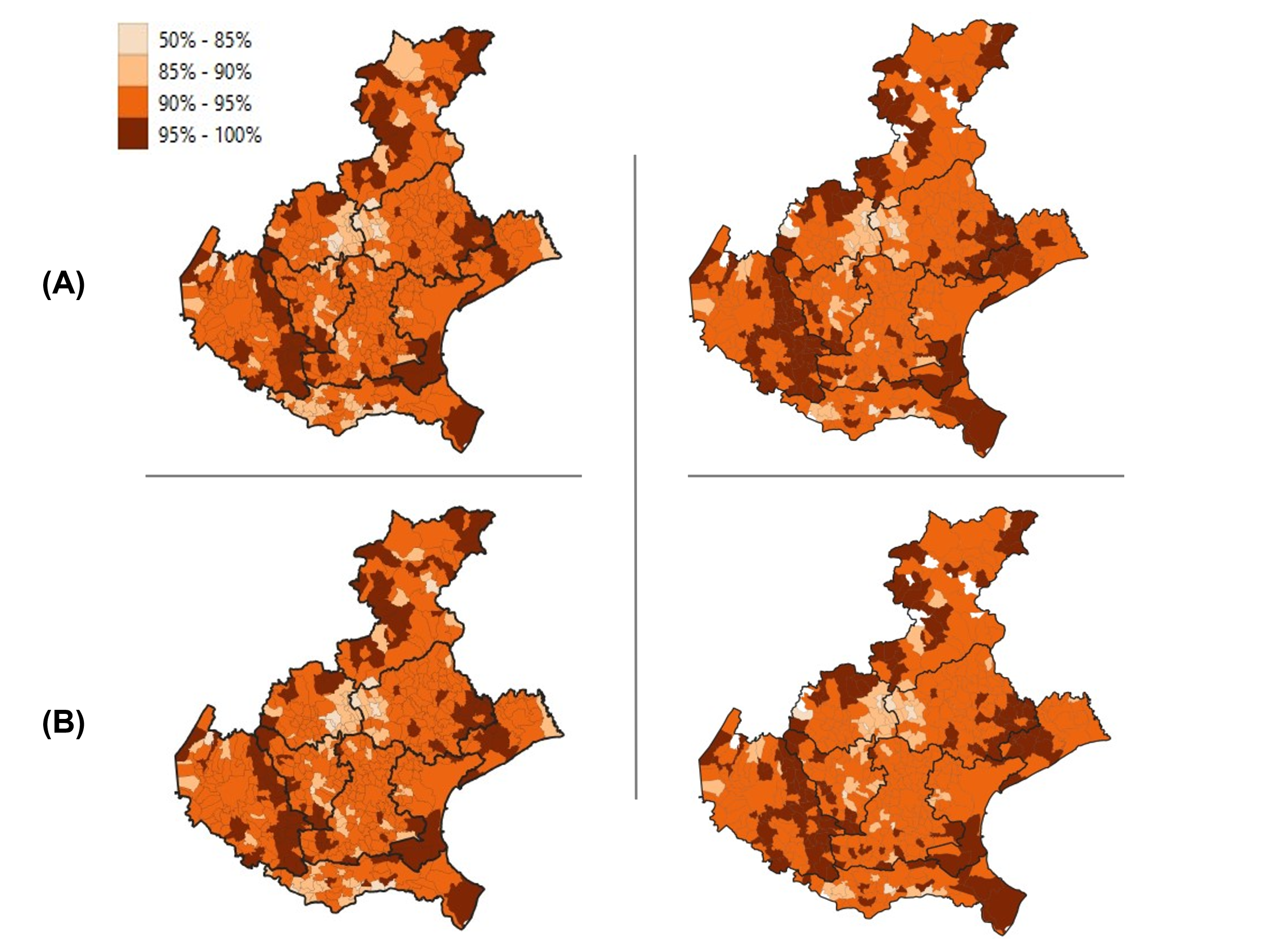


**S6. Percentage of coverage for diphtheria (A) and polio vaccine (B), as per criteria, for individuals aged 1 to 18 years (cohorts 2021-2004); for the municipality of residence**.

Data as of 04/10/2022 (left) and 01/09/2023 (right). Thicker black lines delineate the boundaries of LHAs, while white areas represent municipalities with fewer than five unvaccinated children.


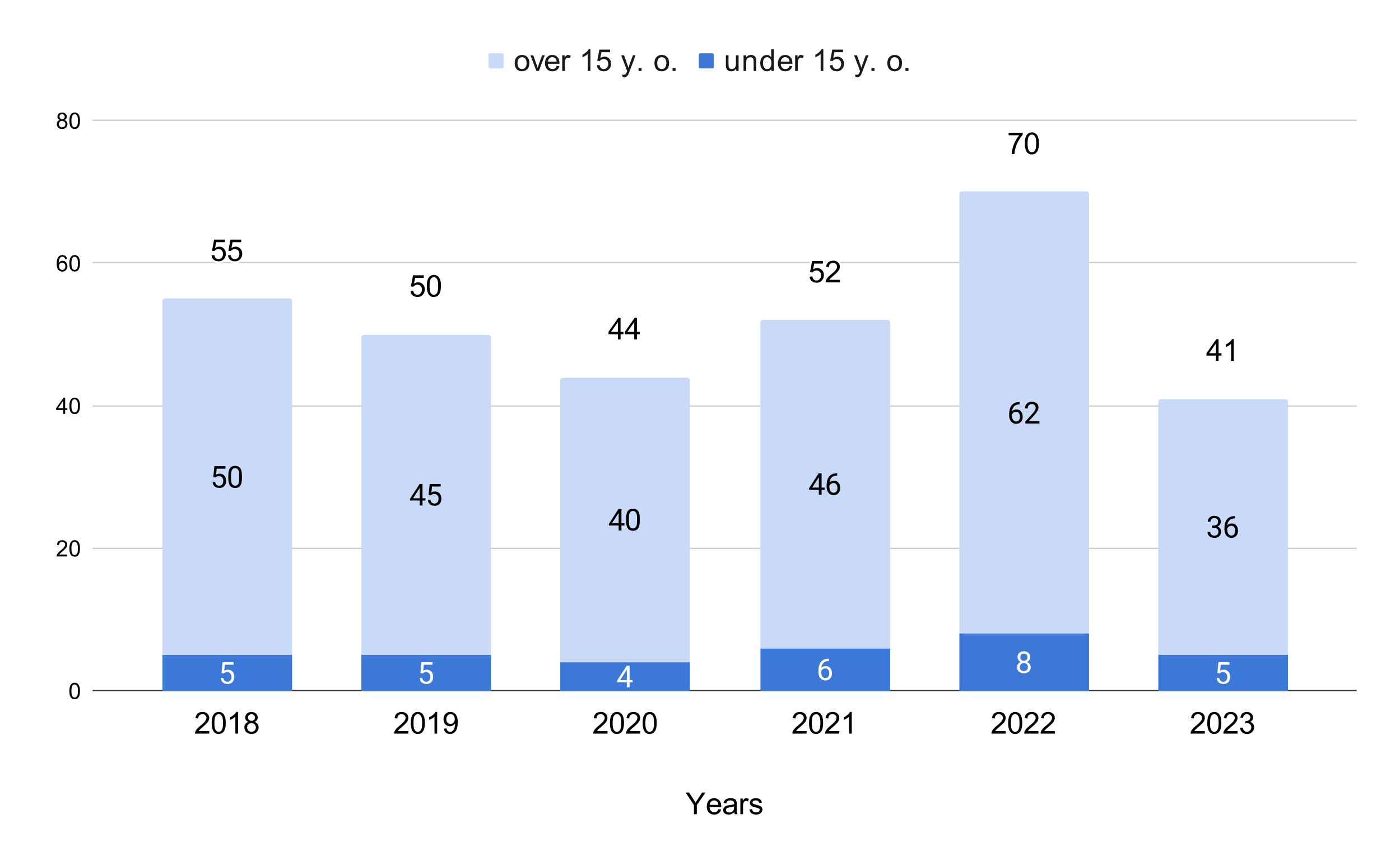


**S7. Number of reported acute flaccid paralysis (AFP) cases in the Veneto Region, sorted by year and age group**

| **Local Health Authority (LHA)** | **Total Population** | **Age 0-14** | **Age 15-64** | **Age 65 and Over** | **Aging Index** | **% Foreign Population** |
| --- | --- | --- | --- | --- | --- | --- |
| LHA 1 | 199,599 | 11% | 62% | 27% | 241 | 5.9% |
| LHA 2 | 878,070 | 13% | 64% | 23% | 169 | 10.1% |
| LHA 3 | 616,323 | 12% | 63% | 25% | 213 | 10.6% |
| LHA 4 | 226,619 | 12% | 64% | 24% | 199 | 9.2% |
| LHA 5 | 232,061 | 11% | 62% | 27% | 250 | 7.8% |
| LHA 6 | 927,111 | 13% | 64% | 23% | 182 | 9.9% |
| LHA 7 | 361,979 | 13% | 64% | 23% | 174 | 7.4% |
| LHA 8 | 488,400 | 13% | 65% | 22% | 169 | 10.4% |
| LHA 9 | 922,291 | 14% | 64% | 22% | 166 | 11.6% |

**S8. The table highlights the demographic distribution across Veneto's local health authorities (LHAs). Legend: Aging Index: Ratio of the population aged 65 and over to the population aged 0-14 (per 100); % Foreign Population: Percentage of residents with non-Italian citizenship out of the total population**.
